# Rheumatoid arthritis, item response theory, Blom transformation, and mixed models

**DOI:** 10.1186/1753-6561-1-s1-s116

**Published:** 2007-12-18

**Authors:** Aldi T Kraja, Jon Corbett, An Ping, Rosa S Lin, Petra A Jacobsen, Michael Crosswhite, Ingrid B Borecki, Michael A Province

**Affiliations:** 1 Division of Statistical Genomics, Washington University School of Medicine, Division of Statistical Genomics, 4444 Forest Park Boulevard, Campus Box 8506, St. Louis, Missouri 63110, USA

## Abstract

We studied rheumatoid arthritis (RA) in the North American Rheumatoid Arthritis Consortium (NARAC) data (1499 subjects; 757 families). Identical methods were applied for studying RA in the Genetic Analysis Workshop 15 (GAW15) simulated data (with a prior knowledge of the simulation answers). Fifty replications of GAW15 simulated data had 3497 ± 20 subjects in 1500 nuclear families. Two new statistical methods were applied to transform the original phenotypes on these data, the item response theory (IRT) to create a latent variable from nine classifying predictors and a Blom transformation of the anti-CCP (anti-cyclic citrinullated protein) variable. We performed linear mixed-effects (LME) models to study the additive associations of 404 Illumina-genotyped single-nucleotide polymorphisms (SNPs) on the NARAC data, and of 17,820 SNPs of the GAW15 simulated data. In the GAW15 simulated data, the association with anti-CCP Blom transformation showed a 100% sensitivity for SNP1 located in the major histocompatibility complex gene. In contrast, the association of SNP1 with the IRT latent variable showed only 24% sensitivity. From the simulated data, we conclude that the Blom transformation of the anti-CCP variable produced more reliable results than the latent variable from the qualitative combination of a group of RA risk factors. In the NARAC data, the significant RA-SNPs associations found with both phenotype-transformation methods provided a trend that may point toward dynein and energy control genes. Finer genotyping in the NARAC data would grant more exact evidence for the contributions of chromosome 6 to RA.

## Background

Rheumatoid arthritis (RA) is a complex disease with a heritability of about 60% as evaluated in twin studies. Univariate qualitative assessments of RA did not reveal a large variation, and quantitative assessment of anti-cyclic citrullinated protein antibodies (anti-CCP), showed a non-normal distribution, although anti-CCP has been considered a better predictor of erosive outcome compared to the rheumatoid factor (RF) IgM [1-4]. In our study we propose the application of item response theory (IRT) and the Blom transformation, respectively, on the qualitative and quantitative variables. Utilizing the data provided by the North American Rheumatoid Arthritis Consortium (NARAC) and the simulated data provided by the Genetic Analysis Workshop 15 (GAW15), we explore the above two statistical methods that transform the phenotypes. The association of the new RA phenotypes in combination with the linear mixed effects (LME) models summarizes the genetic effect of individual SNPs on RA. The significant single-nucleotide polymorphisms (SNPs) in the NARAC real data and from the GAW15 dense SNPs simulated ones on chromosome 6 setting are reported.

## Methods

### Item response theory (multivariate qualitative to quantitative latent variable transformation)

In the application of the IRT method in handling multivariate phenotypic data, it was assumed that each individual classified with RA had a latent RA variable. Such a latent feature for RA was the result of a statistical summary of information from nine observed risk factors. These factors were qualitative anti-CCP, qualitative IgM, gender, smoking status, and five severity classes of left and right hand erosions for RA, which were transformed to individual severity dummy variables as 1/0 for each corresponding combined class (see Data Input). Applying IRT, each member of the sample was assigned a latent (*z*_*i*_) score. Our analysis is constraint to one latent variable *z*_1*i*_. The probability density function of *y*_*i *_given the latent variable *z *can be written as $p(y_{i}|z,x,\theta_{i},\emptyset_{i})=e\left\{\frac{y_{i}\theta_{i}-\beta_{i}(\theta_{i})}{\alpha_{i}(\emptyset_{i})}+d_{i}(y_{i},\emptyset_{i})\right\}$, where *θ*_*i *_and ∅_*i *_are the location and dispersion parameters, respectively. In the IRT, the item characteristic curves represent the positive response probability of each risk factor *j *relative to the latent RA score. Formally, this relationship is expressed as $p\left(y_{ij}=1|z_{i}\right)=\frac{1}{1+e\left\{-\alpha_{i}(z_{i}-\beta_{i})\right\}}$, and graphically it represents the importance of each risk factor toward the latent RA. The discrimination parameter *α*_*i *_is viewed as how well a risk factor discriminates among subjects with opposite extreme latent RA. The difficulty parameter *β*_*i *_characterizes the difficulty level of individual risk variables. In this study we applied the two-parameter IRT model (*α*_*i*_, *β*_*i*_), which was constrained to one latent variable (*z*_*i*_). Software for IRT analysis in R language is developed by Rizopoulos (ltm package version 0.5–1 for Linux OS and R version 2.3.0) [5,6].

### Blom score (quantitative univariate to quantitative rank score transformation)

In the second method we used the Blom transformation. Blom scores represent rank approximations of the exact order of a normal distribution. In the group of rank transformed quantitative variables, one extracts a Blom score by applying the following formula on the anti-CCP.

$Blom_{i}=\frac{\phi_{i}^{-1}(r_{i}-\frac{3}{8})}{n+\frac{1}{4}}$, where *ϕ*_*i*_^-1 ^is the inverse of cumulative normal function, *r*_*i *_is the rank of observation *i*, and *n *is the number of non-missing observations. The Blom scores of anti-CCP represented a better normal distribution than the original values. Software for the Blom transformation is available within the PROC RANK of SAS, v 9.1.3 for Linux OS.

### Linear mixed effects models (association tests)

The LME model used in our study to test the association between an SNP and changes in the response variable (IRT RA latent variables/anti-CCP Blom transformed variables) follows in a matrix form: ***Y ***= ***XB ***+ ***ZU ***+ **ε**, where ***Y ***is an *m *× 1 vector of responses; ***X ***is an *m *× *p *design matrix of the fixed effects; ***B ***is the parameter *p *× 1 vector of fixed effects; ***Z ***is an *m *× *q *incidence matrix of random effects, and ***U ***is a *q *× 1 vector of random effects with *E*(***U***) = **0**, and covariance matrix ***G***; ***0 ***is an *m *× 1 vector of random effects with *E*(***0***) = **0 **and covariance matrix ***R***. In the fixed effects we included SNP genotypes recoded as additive effects (-1 for one homozygote, 0 for the heterozygote, and 1 for the other homozygote genotype), and gender. In the random effects we included the family identification number. We tested whether the SNPs additive effects are different from zero, and especially we identified the highest significances, considering that multiple comparisons as well as correlated SNPs tests because of linkage disequilibrium are present. The LME in SAS (v. 9.1.3 for Linux OS) was applied with PROC MIXED using the option for EMPIRICAL variance, which computes the estimated variance-covariance matrix of the fixed-effects parameters by using the following asymptotically consistent estimator (XV‵−1X)−(∑i=1sXiV‵i−1∈i∈i`V‵i−1Xi)(XV‵−1X)`, where var(***Y***) = ***V ***= ***ZGZ***' + ***R ***[7].

Results of association tests in 50 replications of the simulated data were analyzed with two definitions: **sensitivity**, defined as the proportion of the true causative SNPs that had a positive association result (*p*-value < 0.05); and **specificity**, defined as the proportion of true non-causative SNPs that had non-significant associations (*p*-value ≥ 0.05).

### Data input

NARAC phenotypes in our analysis included anti-CCP, rheumatoid factor (RF), gender, severity of left and right hand side erosions on a scale of 1 to 5 (severityLH and severityRH, respectively), whether or not the subject smoked cigarettes (HxCigN). Qualitative dummy variables were created for each of the following variables, where the subject with anti-CCP > 49 [8], RF > 170 (to capture approximately the RF upper quartile in a very skewed RF distribution); smokers (HxCigN = 2); gender (if female); and whether severityLH or severityRH was 1, 2, 3, 4, or 5 were considered as affected. In the corresponding nine dummy variables (accp, igm, smok, sex, sev1, sev2, sev3, sev4, and sev5) affection was codes as 1, unaffected as 0, otherwise as missing. The same nine dummy variables were created also for the RA GAW15 simulated data. These dummy variables were analyzed individually for the two sources of data with the ltm package to extract the corresponding RA latent factors. Also, the anti-CCP variables were independently Blom-transformed within groups defined by gender and each age decade. LME model was performed to evaluate association of these traits, with 404 Illumina SNPs on chromosome 6 for NARAC data, and with 17,820 dense simulated SNPs on chromosome 6 for 50 replications on the simulated GAW15 data. In the NARAC data the sample size was 1499 subjects and 757 families, and 1340 subjects and 757 families for anti-CCP Blom transformation and IRT latent variable, respectively. In the simulated data the sample contained a mean of 3497 ± 20 subjects and 1500 nuclear families for both traits.

## Results

**Figure 1:**
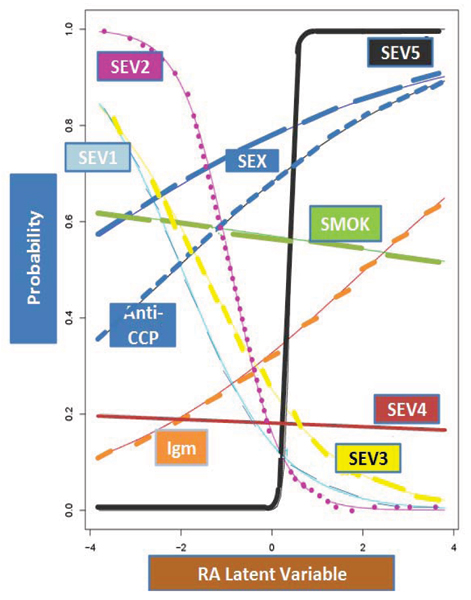
NARAC data item characteristic curves (see Methods and Results for details).

We applied two new methods to transform the RA phenotypes and performed linear mixed effects models association tests. The IRT latent variables were created as a combination of nine predictors, which were considered as binary classifiers of RA. Figure 1 shows the NARAC data item characteristic curves. Risk variable severity 5 (sev5) was the most informative item for separating subjects with positive and negative latent RA variable (high level of discrimination). Gender represented an easy item, because the probability was high for a negative or positive RA latent response. The anti-CCP item had a relatively similar property of discrimination as IgM because it differentiated relatively well among subjects having RA latent variable below the item location and those RA latent variable above the item location. By contrast sev1, sev2, sev3, and sev4 showed a negative discrimination, which means that these risk factors were less reliable in identifying subjects with positive RA latent variable. In the simulated data, we considered 100 replications only to estimate the IRT parameters (item discrimination and difficulty). The mean discrimination parameters of anti-CCP and IgM had similar values with the NARAC original data, but the difficulty parameters were quite variable in the simulated data. For anti-CCP, the difficulty parameter was -2.1 in the original data, and in the simulated data the median was -2.29 with a mean standard deviation of 250; for IgM the difficulty parameter was 2, and in the simulated data the median was -3.6 with a mean standard deviation of 85.

In the simulated data, out of 50 replications, the association of SNP1 (locus DR) with the IRT latent variable based on LME was 24% sensitive and 94.79% specific. SNP2 and SNP3 together (Locus D) had a lower sensitivity (7%), but the same specificity as SNP1. When a larger region (~500 kb) was assumed as the corresponding genes, for GENE1 (corresponding to SNP1) the sensitivity was 7.3%, for GENE2 (corresponding to SNP2 and 3) the sensitivity was only 4.8%. In contrast, the sensitivity of the association of the Blom transformed anti-CCP with SNP1 was 100%. For SNP2 and SNP3 the sensitivity was 6%. Also for GENE1 the association sensitivity with anti-CPP Blom transformed was 46.2%.

Single SNPs association results with *p*-values less than 0.05 from the NARAC data are shown in Table 1. Out of 17 anti-CCP Blom-transformed SNPs and 25 SNPs for IRT latent variable significant associations, only two (rs910563 and rs923459) overlap between the two responses analyzed.

**Table 1:** NARAC data, additive model *p*-values and effects for the anti-CCP Blom-transformed and the IRT latent variable data^a^

	Anti-CCP Blom-transformed					IRT latent variable				
		
No.	SNP	*p*-value	Additive effect	STD	bp	SNP	*p*-value	Additive effect	STD	bp
1	rs189512	0.00067	-0.14680	0.04294	6923632	rs2876143	0.00816	0.07944	0.02993	8928623
2	rs214527	0.01890	-0.11800	0.05014	18399329	rs1863995	0.04430	-0.05370	0.02665	13565596
3	rs1402405	0.02528	0.08417	0.03755	24198476	rs736794	0.04758	-0.04813	0.02425	41044688
4	rs1224485	0.02145	0.08928	0.03874	37043628	rs1009130	0.00301	0.08090	0.02716	58068726
5	rs1738240	0.01339	0.10040	0.04051	38810588	rs977382	0.02981	0.06241	0.02866	62057200
6	rs533393	0.00897	-0.10350	0.03951	81645240	rs1040802	0.01273	-0.06005	0.02403	63469278
7	rs1180237	0.01758	0.09720	0.04085	83939524	rs2076874	0.00397	0.06580	0.02276	64150527
8	**rs910563**^b^	0.01025	0.10250	0.03983	**88054481**	rs1630182	0.02261	-0.05252	0.02298	64205048
9	rs1051131	0.04853	0.07951	0.04024	88217142	rs1370436	0.03343	0.05014	0.02352	74520012
10	rs2144363	0.02680	0.19480	0.08778	92037502	**rs910563**	0.03853	0.04794	0.02312	**88054481**
11	rs6454855	0.04535	0.07830	0.03906	92071536	rs2610715	0.01464	-0.05937	0.02425	89260502
12	rs716192	0.04829	-0.08171	0.04131	96557287	rs240153	0.04093	-0.04727	0.02307	101112733
13	rs1321807	0.02545	0.08678	0.03876	117730562	rs239229	0.02728	-0.05129	0.02318	101151573
14	rs941815	0.04541	-0.08273	0.04128	123901148	rs239189	0.03231	0.04970	0.02317	101174385
15	rs839556	0.04100	0.08086	0.03950	142852448	rs1158747	0.03314	-0.05161	0.02417	112546220
16	**rs923459**	0.00991	0.10150	0.03927	**159471138**	rs763075	0.00505	0.06554	0.02330	122192096
17	rs294882	0.02322	-0.09232	0.04059	159512287	rs1033540	0.04603	-0.04924	0.02463	131717872
18						rs225604	0.00643	0.06488	0.02373	142433136
19						rs1931992	0.00209	0.07516	0.02433	142581777
20						rs2151913	0.03291	-0.05082	0.02377	150161633
21						rs718527	0.01903	-0.05770	0.02454	152501127
22						rs231958	0.01449	0.06105	0.02490	155786394
23						**rs923459**	0.01838	0.05568	0.02356	**159471138**
24						rs409359	0.04983	-0.04526	0.02303	159854284
25						rs916331	0.01358	0.05892	0.02380	167379847

^a^See the Discussion paragraph for details on the genes that have significant SNPs part of this study. bBold text indicates significant SNPs replicated in the association tests for two different RA outcomes.

## Discussion

Of 404 Illumina SNPs typed on chromosome 6, none of the significant SNPs passed the 0.0001 Bonferroni threshold. Because such threshold is considered to be conservative, in Table 1 we show the original significant *p*-values of the SNPs associated with the two explored phenotypic transformations, Blom and IRT. Two SNPs, namely rs910563 and rs923459, overlapped as significant in both analyses. rs910563 is in an intron in the *C6orf163 *gene. Its function is unknown. Significant SNPs were also located in other interesting genes. Of them, rs533393 is in an intron in the *DNAH8 *gene, which is one of the dynein heavy chains responsible for force production and ATPase activity. It contains a highly conserved catalytic domain. The rs1180237 is in an intron in the *ME1 *gene, which encodes a cytosolic, NADP-dependent enzyme that generates NADPH for fatty acid biosynthesis. Another SNP, rs1051131, although located on the exon 8 region of gene *SLC35A1*, which is a solute carrier family 35 (CMP-sialic acid transporter), member A1, is untranslated. SNP rs716192 is located in the *FUT9 *(fucosyltransferase 9) gene. rs1321807 is part of the gene *ROS1*. Its protein may function as a growth or differentiation factor receptor. SNP rs941815 is part of the gene *TRDN*. Experimentally it is proposed that one of the Triadin genes overexpression blocks excitation-contraction coupling in rat skeletal myotubes. The SNP rs294882 is located in the gene *FNDC1 *(fibronectin type III). Fibronectin is a key extracellular matrix protein that not only provides a substrate for cell anchorage but also serves as a regulatory protein in processes such as cell adhesion, motility, differentiation, and proliferation.

Amos et al. [9] documented a very significant linkage peak on chromosome 6, and implicated the major histocompatibility complex in risk for RA. In the GAW15 simulated data this important region was marked with the SNP1 at locus HLA-DRB1. Our association results found that the simulated SNP1 associated significantly 100% of the time with the Blom-transformed anti-CCP values, but only 24% with the IRT latent variable (Table 2, and Figure 1). We feel that this large difference in the sensitivity of the IRT approach versus the Blom transformation in the simulated data may be influenced to some extent by the simulation settings. Also the fact that Blom approach is applied on a quantitative trait and the IRT approach is performed on qualitative traits contributes in the power differences of these analyses.

**Table 2:** Sensitivity and specificity of three SNPs associations with two different phenotypes (50 replications)

	Sensitivity (%)					Specificity (%)				
		
Traits(s) transformation	SNP1^a^	SNP2&3^b^	SNP1-3	GENE1^c^	GENE2	SNP1	SNP2&3	SNP1-3	GENE1	GENE2
IRT latent variable	24.00	7.00	12.70	7.30	4.80	94.79	94.79	94.79	94.80	94.78
Anti-CCP Blom transformation	100.00	6.00	37.30	46.20	10.10	93.21	93.20	93.21	93.42	93.21

^a^SNP1 is located at 32,484,648 bp and labeled as m3437.

^b^SNP2&3 flank the location 37,233,784 bp and labeled as m3916 and m3917.

^c^GENE1 and GENE2 represent, respectively, a region ~500 kb around SNP1 (assumed as locus DR) and around SNP2 and SNP3 (Locus D).

## Conclusion

From our findings with the simulated data, we assume that the Blom-transformed NARAC anti-CCP data can be a more reliable phenotype in the analysis to identify true significant associations. Amos et al. documented the highest chromosome linkage peak with two SNPs, rs169679 and rs11908. Although these SNPs were present in our analysis, neither were significant. It is possible that differences stand in the facts that two analyses are different (linkage vs. association) and different traits were considered. In conclusion, our results provide a compelling trend that may point toward dynein and energy control genes involvement in the RA. In the future, denser genotyping may provide more exact evidence for chromosome 6 and its contribution to RA.

## Competing interests

The author(s) declare that they have no competing interests.
